# How COVID-19 Pandemic Changed Consumption of Fruits and Vegetables by Older Adults

**DOI:** 10.1093/geroni/igab046.2706

**Published:** 2021-12-17

**Authors:** Lillie Monroe-Lord, Azam Ardakani

**Affiliations:** 1 University of the District of Columbia, Washington, District of Columbia, United States; 2 Howard University, Howard University, District of Columbia, United States

## Abstract

This study aims to determine the changes in consumption of fruits and vegetables of older adults before and since the COVID-19 pandemic. The data collection was administered by Qualtrics through an online survey conducted in August and September 2020. The total participants were 10,050. Differences between consumption of fruits (fresh, canned, frozen) and vegetables (carrots, sweet potatoes, broccoli, spinach) before and since COVID-19. Date were analyzed using the Wilcoxon’s signed-rank test. Among participants, 5,767 females (57.4%) and 4,283 males (42.6%) and the average age of 62.09 (SD=11.22). 7.1% were Asian (N=701), 4.3% were Hispanic (N=429), 14.1% were African Americans (N=1393), and 74.5% were White (N=7,390). For total participants, consumption of fruits decreased significantly (p<0.001) since COVID-19. The decrease in consumption of fruits was larger in females (p<0.001) than males (p=0.026). It is likely because consumption of fruits by males was already low before the pandemic at 27% of the amount consumed by females. The decrease in consumption of fruits was not statistically significant in Asian (p=0.096) and African American (p=0.07), but significant in Hispanic (p=0.008) and White (p<0.001) participants. African American and Hispanic participants consumed a lower number of fruits before the pandemic compared to Asian and White participants. Consumption of vegetables had no significant change since COVID-19 for total participants regardless of gender and race. This study reported a significant decrease in the consumption of fruits, but not vegetables by older adults since COVID-19 pandemic.

